# Probing Mechanoregulation of Neuronal Differentiation by Plasma Lithography Patterned Elastomeric Substrates

**DOI:** 10.1038/srep06965

**Published:** 2014-11-07

**Authors:** Ki-Hwan Nam, Nima Jamilpour, Etienne Mfoumou, Fei-Yue Wang, Donna D. Zhang, Pak Kin Wong

**Affiliations:** 1 Department of Aerospace and Mechanical Engineering, The University of Arizona, Tucson, Arizona 85721, USA; 2 Centre for Analytical Instrumentation Development, The Korea Basic Science Institute, Deajeon305-806, Korea; 3 The Key Laboratory for Complex Systems and Intelligence Science, The Institute of Automation, Chinese Academy of Sciences, Beijing, China; 4 Department of Pharmacology and Toxicology, The University of Arizona, Tucson, Arizona. 85721, USA

## Abstract

Cells sense and interpret mechanical cues, including cell-cell and cell-substrate interactions, in the microenvironment to collectively regulate various physiological functions. Understanding the influences of these mechanical factors on cell behavior is critical for fundamental cell biology and for the development of novel strategies in regenerative medicine. Here, we demonstrate plasma lithography patterning on elastomeric substrates for elucidating the influences of mechanical cues on neuronal differentiation and neuritogenesis. The neuroblastoma cells form neuronal spheres on plasma-treated regions, which geometrically confine the cells over two weeks. The elastic modulus of the elastomer is controlled simultaneously by the crosslinker concentration. The cell-substrate mechanical interactions are also investigated by controlling the size of neuronal spheres with different cell seeding densities. These physical cues are shown to modulate with the formation of focal adhesions, neurite outgrowth, and the morphology of neuroblastoma. By systematic adjustment of these cues, along with computational biomechanical analysis, we demonstrate the interrelated mechanoregulatory effects of substrate elasticity and cell size. Taken together, our results reveal that the neuronal differentiation and neuritogenesis of neuroblastoma cells are collectively regulated via the cell-substrate mechanical interactions.

Human neuroblastoma is a pediatric tumor of the neural crest and heterogeneous cell composition is observed in both tumors and tumor-derived cell lines^1^. Neuroblastoma is one of the few malignancies demonstrating spontaneous differentiation and regression to a benign state^2^, and is capable of self-renewal and generating partially differentiated progenitor cells, characteristics of cancer stem cells^1^. Neuronal differentiation of neuroblastoma cells can be induced by retinoic acid^3^. The neuronal phenotype (N-type) is characterized by highly refractive cells, reduced cell growth, and the formation of distinct neurites (neuritogenesis). The neuronal cells adhere weakly to the substrate and grow as clumps of cells. For instance, SH-SY5Y, a neuroblastoma cell line, is an in *vitro* model for investigating the early stages of neuronal differentiation and neural tissue engineering strategies^4^,^5^,^6^,^7^. Differentiation of SH-SY5Y induced pharmacologically has also been studied as a model of dopaminergic neurons for Parkinson's disease research^8^. Increased understanding and the ability to induce neuroblastoma differentiation may have important implications in regenerative medicine and disease therapeutics.

Cell behavior, such as development and regeneration, is often influenced by cell-cell or cell-microenvironment interactions^9^,^10^. Mechanical cues are known to modulate the differentiation of neuroblastoma and stem cells. For instance, neurite outgrowth of neuroblastoma cells can be promoted and guided by substrate stiffness^11^,^12^,^13^ and spatial pattering^14^,^15^,^16^. Notably, a combination of multiple mechanical factors may modulate cell behavior (e.g., differentiation of human mesenchymal stem cells) in a complex manner^17^,^18^. However, there is a paucity of knowledge on the relationship between different mechanoregulatory factors and the process by which neuronal differentiation is regulated by these mechanical cues remains poorly understood. Since cells naturally experience multiple mechanical cues in the microenvironment, a comprehensive investigation on the effects of these factors will improve our fundamental understanding in neuronal differentiation and may facilitate the design of translational biomaterials and regenerative medicine in the future.

Cell patterning techniques, such as contact printing and photolithography, are available for biomechanical studies^19^. To allow for simultaneous control of the substrate elasticity and geometric constraint, cell patterning techniques should be compatible with substrate materials that have tunable elasticity. Another important consideration in selecting a patterning technique is the stability of the patterns. Cell-mediated degradation and physical desorption of the extracellular matrix and cell repellent molecules can occur in long term studies that take days to weeks, such as tissue morphogenesis and cell differentiation^20^. To address these issues, plasma lithography^21^,^22^,^23^ has been developed for investigating tissue development, intercellular communication, and migration in confined environments^24^,^25^,^26^,^27^,^28^. The technique has rapid processing time (10 min), long-term stability (more than 2 months)^28^, and high spatial resolution (100 nm)^22^. Plasma lithography is one of a few techniques specialized for patterning polymeric materials, which have a wide range of achievable mechanical and biochemical properties. Nevertheless, the potential of plasma lithography in patterning elastomeric materials, such as polydimethylsiloxane (PDMS) that are widely-used in microfluidic cell culture, cell traction force measurement, and investigation of the effects of substrate stiffness^29^,^30^,^31^,^32^, has not been demonstrated. Plasma lithography patterning on elastomeric substrates will enable simultaneous control of substrate elasticity and geometric constraints, allowing for the investigation of neuritogenesis and neuroblastoma differentiation.

In this study, we present plasma lithography of elastomeric materials to simultaneously control substrate elasticity, geometric constraint, and cell size for elucidating the influences of the cell-substrate mechanical interactions on the differentiation of neuroblastoma cells. We perform plasma lithography patterning on PDMS substrates with adjustable elasticity by controlling the PDMS crosslinker concentration. The plasma lithography creates stable patterns throughout the duration of the experiment and allows us to investigate the effects of the geometric confinement on neuronal sphere differentiation, which requires several days. We apply the approach to study retinoic acid-mediated neuronal differentiation and study the influences of mechanical factors on focal adhesion, neurite elongation, and morphology of SH-SY5Y neuronal spheres. A computational biomechanical model is also developed to interpret the interrelated effects of geometric constraint, substrate stiffness, and cell size on the cell-substrate mechanical interactions.

## Results

### Cellular patterning by plasma lithography on elastomeric substrates

**Figure 1:**
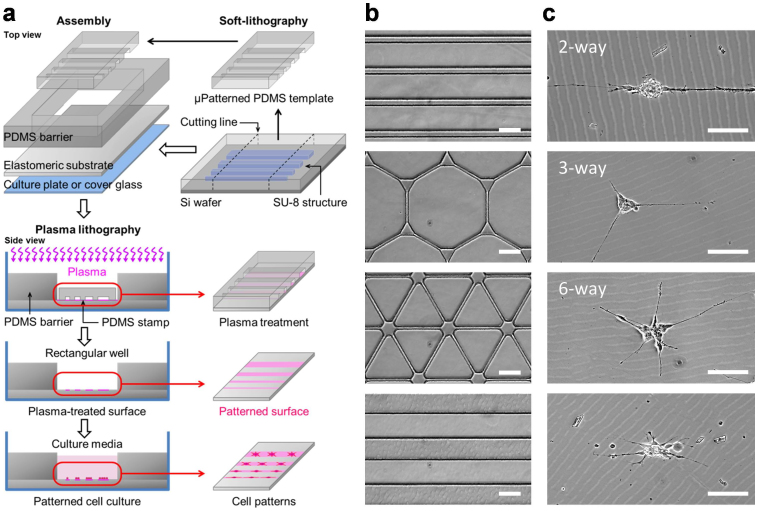
Plasma lithography patterning of neuroblastoma cells. (a) Schematics of plasma lithography for confining neuroblastoma cells on elastomeric substrates. PDMS templates were fabricated using photolithography and PDMS molding. Cell patterns were created on substrates by atmospheric plasma with selective shielding by the PDMS template. The cells were maintained in the patterned region by a PDMS well. Retinoic acid was introduced to induce neuronal differentiation. (b) PDMS templates with different patterns for cell confinement. (c) Bright-field images of neuronal spheres confined in plasma lithography patterned regions. The images were captured after culture of neuroblastoma cells for 6 days. Scale bars, 100 μm.

Plasma lithography was applied to create surface patterns for geometric confinement of neuroblastoma cells. Plasma lithography patterning was performed by first fabricating a PDMS template followed by selective plasma treatment of the elastomeric substrate with the template (Fig. 1a). The template spatially shielded the substrate from plasma treatment, and consequently only the unshielded areas were exposed and functionalized by plasma, creating patterns for cell attachment. To demonstrate the applicability of plasma lithography on PDMS substrates, line and network patterns were designed for studying neuronal differentiation. Figures 1b–c show patterns of PDMS templates and neuroblastoma cells confined in plasma-treated, hydrophilic areas of the substrates. The smallest pattern width for cell attachment was 10 μm. The value was larger than other cell types, e.g. fibroblasts, endothelial cells and myocytes (~1 μm)^24^,^25^,^26^,^27^,^28^. The large pattern width required for cell adhesion is consistent with the weak adhesion property of the N type cells. For smaller patterns, the cells either did not attach initially, or detached from the substrate after a short time. With an appropriate cell density (70–210 cells/mm^2^), and with retinoic acid treatment, the neuroblastoma cells formed neuronal spheres with neurite outgrowth (Supplementary Fig. S1). The neurites were extended along the plasma-treated patterns and did not go off the patterns in the duration of the experiment (up to 15 days). Cell attachment and neurite elongation could be guided through two-, three-, and six-way connected networks (Fig. 1c). Line patterns with different widths were designed in this study to systematically investigate the effects of geometric constraints on neuronal differentiation. These results demonstrate that plasma lithography is capable of patterning neuroblastoma cells and neuronal spheres, and provides an effective method for investigating geometric control of neuronal differentiation.

### The influence of substrate elasticity on neuronal differentiation

**Figure 2:**
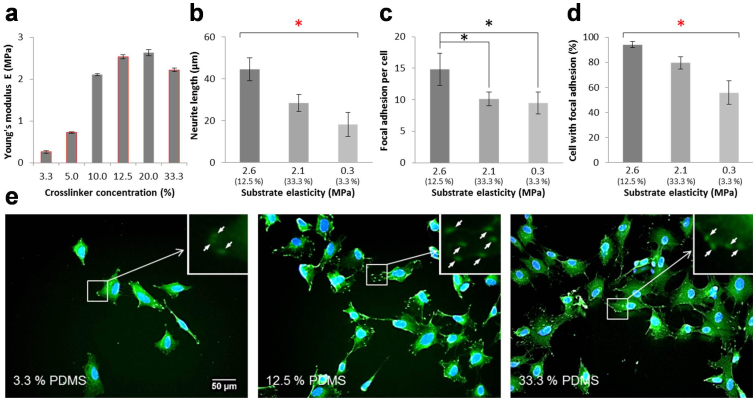
Differentiation of neuroblastoma cells on PDMS substrates with different elasticities. (a) The Young's modulus of PDMS with crosslinker concentrations from 3.3% to 33.3%. Error bars indicate the standard deviation of ten measurements on each condition. (b) The length of neurites on PDMS with different elasticities. (c) The number of focal adhesions for cells on substrates with different elasticities. (d) The fraction of cells exhibiting observable focal adhesions. To define focal adhesion cells, a cell with at least 3 distinguishable focal adhesions qualified. Error bars represent standard deviations of 3 replicates (n > 90 for each group from three independent experiments). Statistical analysis was carried out by Student's t-test (black stars) and two-way ANOVA test followed by post-hoc Duncan test (red stars). A P-value of p < 0.05 was considered significant. (E) Representative fluorescence images of retinoic acid-treated SH-SY5Y cells cultured on PDMS with (left) 3.3%, (middle) 12.5%, and (right) 33.3% of crosslinker. Cells were stained with anti-vinculin antibodies (green) and DAPI (blue). White arrows in inserts indicate visible focal adhesions.

To investigate the effects of substrate elasticity on neuronal differentiation, the elastic moduli of PDMS with different crosslinker concentrations were characterized. Figure 2a shows the Young's modulus of plasma-treated PDMS measured by atomic force microscopy. The stiffness increased with the crosslinker concentration at low values and maximized between 12.5% and 20.0%. The elasticity decreased with further increases in the crosslinker concentration (e.g., 33.3%). The measured elasticities were in good agreement with previous studies^33^. No significant differences (p > 0.1) were observed between samples with and without plasma treatment. According to the measurements, 5.0%, 12.5%, and 33.3% PDMS with elasticities of 0.7 MPa, 2.6 MPa, and 2.1 MPa were chosen in this study. As a reference, the Young's modulus of polystyrene tissue culture plates was 2.8 GPa^34^.

The morphology and the cell-substrate interaction of neuroblastoma cells on PDMS with different elasticities were characterized. A low density cell culture was applied to facilitate visualization and data analysis at the single cell level. It was observed that the average length of neurites increased with the substrate elasticity (Fig. 2b). Moreover, the number of focal adhesions and the percentage of cells with detectable focal adhesions increased with the substrate stiffness (Fig. 2c and d). Figure 2e shows representative images of neuroblastoma cells on PDMS with different elasticities. Notably, the neurite outgrowth and focal adhesion formation correlated with the elasticity, instead of the crosslinker concentration, supporting the notion that the observed effects were triggered mechanically. The dependence of elasticity on neuronal differentiation is consistent with a previous study using polyacrylamide hydrogel^12^ and supports the applicability of PDMS for investigating the mechanoregulation of neuronal differentiation.

### The effects of geometric constraints on neuronal differentiation

**Figure 3:**
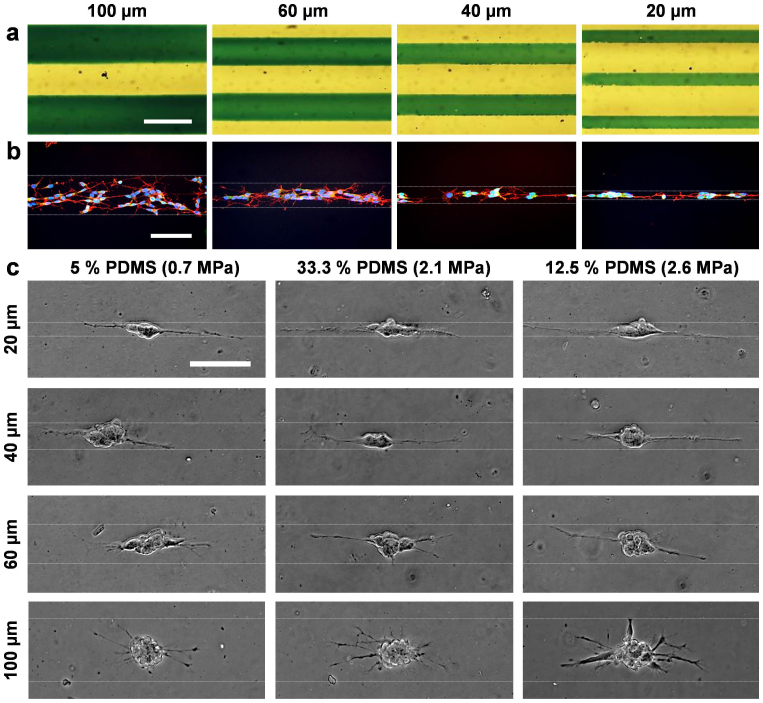
Geometric confinement of neuroblastoma cells. (a) Characterization of the surface patterns by wetting of aqueous green dye solution. (b) Neuroblastoma cells were stained with phalloidin for F-actin (red), anti-vinculin-FITC antibodies for focal adhesions (green), and DAPI for nuclei (blue). White dotted lines indicate regions patterned by plasma lithography. The initial seeding density was 250 cells/mm^2^. (c) Bright-field images of neuroblastoma cells confined in patterns with different widths on PDMS substrates. Cell seeding density on each substrate was 70 cells/mm^2^. Images are representative of three independent experiments. Scale bars, 100 μm.

**Figure 4:**
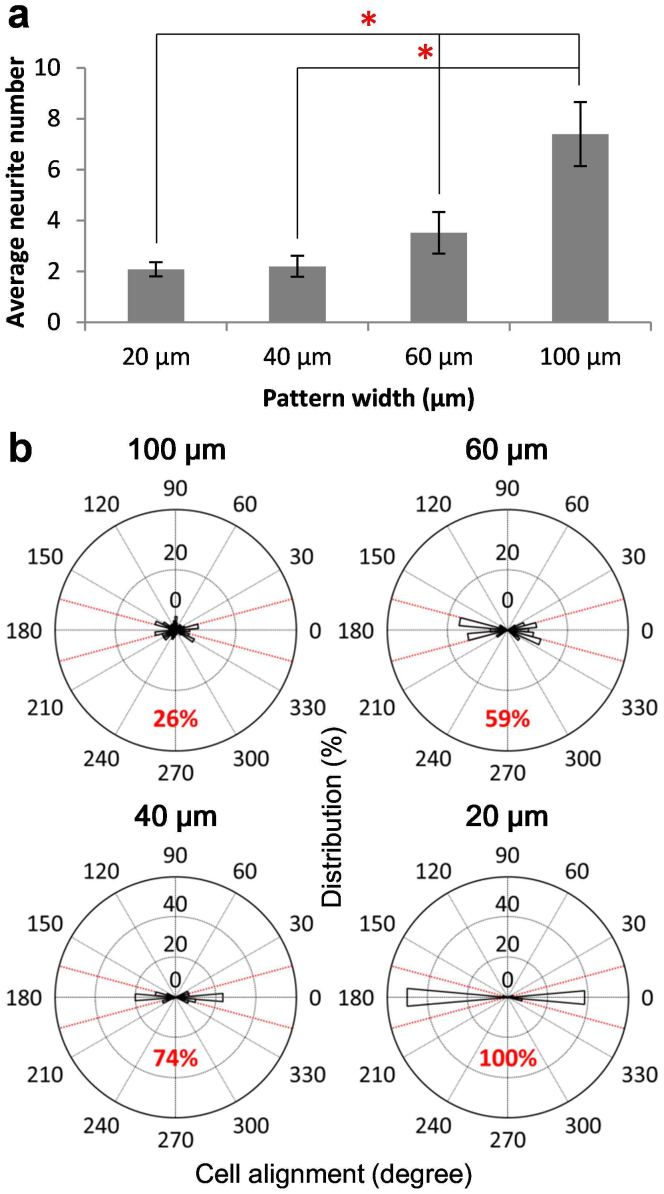
Morphological changes in neuronal differentiation with geometric constraints. (a) The average number of neurites for neuronal spheres patterned on different patterns. Error bars represent standard deviations of 3 replicates (n > 47 for each group). Significance was assumed when p < 0.05 (* in red). (b) Cell alignment on different patterns. The portion of the elongation directions within ±15 degree from the patterns (red dot lines) representing parallel directionality is highlighted in each graph (n > 153 for each condition).

To investigate the effects of geometric constraints on neuronal differentiation, line patterns with different dimensions were created on PDMS. Polystyrene tissue culture plates were applied as control. The dimensions of the patterns were verified by microscopic inspection of food dye solution wetting on plasma-treated areas and cells confined in the patterns (Fig. 3a and b). The neuroblastoma cells were cultured with an initial seeding density of 70 cells/mm^2^ and differentiated on patterns of 20 (23.3 ± 0.4), 40 (41.5 ± 0.4), 60 (62.3 ± 1.1), and 100 (101.7 ± 0.6) μm width created on 5%, 12.5%, and 33.3% PDMS (Fig. 3c). Neuronal spheres with neurite outgrowth were formed in all patterns and substrate elasticities. Figure 4 shows the number of neurites and the directionality of neurite elongation along the line patterns with different widths. The number of neurites increased with the pattern width (Fig. 4a). Approximately 8 neurites on average were elongated from the neuronal spheres pattered on 100 μm width patterns, while only ~2 neurites were observed on 20 μm width patterns. Furthermore, neurites were confined along the pattern direction as the pattern width decreased (Fig. 4b). The percentage of neuroblastoma cells aligning within ±15 degrees (red dotted lines) from the patterns was measured (Fig. 4b, numbers in red). All neurites were aligned within 15 degrees with the pattern on 20 μm lines. Only 26% of neurites were aligned on 100 μm width patterns. Our results demonstrate that neurite outgrowth can be regulated by geometric constraints during neuronal differentiation.

### Interrelated biomechanical effects of elasticity and geometric constraint

**Figure 5:**
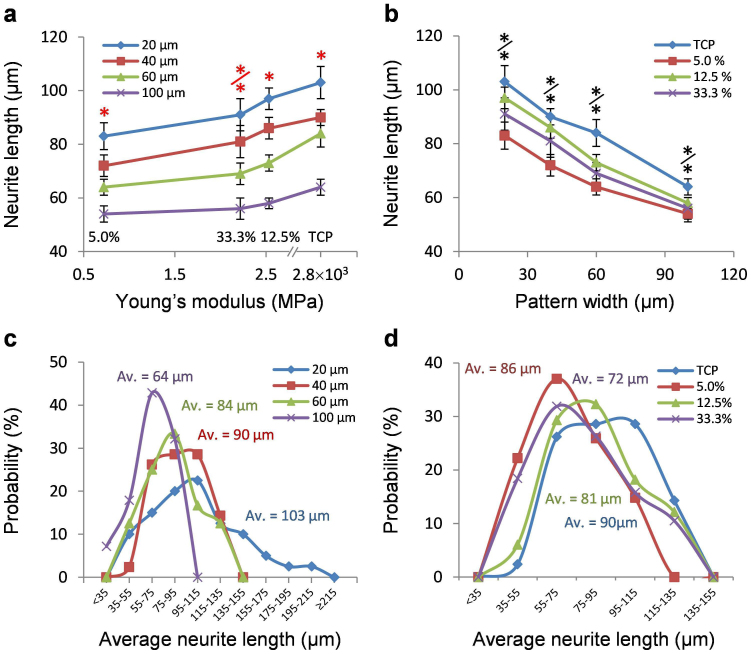
Interrelated effects of geometric constraint and substrate elasticity on neuronal differentiation. (a–b) The average length of neurites for neuronal spheres on substrates with different (a) elasticities and (b) pattern widths. Error bars represent standard deviations of 3 technical replicates (n > 153 for each condition from three independent experiments). Statistical analysis was carried out by Student's t-test (black stars) and two-way ANOVA test followed by post-hoc Duncan test (red stars). Significance was assumed when p < 0.05 (*/* (red): among 20 or 40, and 60, 100 μm; */* (black): between two groups of tissue culture plates vs. 33.3% and 12.5% vs 5.0%). (c–d) Distribution of neurite lengths on substrates with different (c) pattern widths and (d) elasticities. The average length of neurites for each condition is indicated on the curve.

The effects of substrate elasticity and pattern dimension were analyzed simultaneously to explore their relationship on the differentiation of neuronal spheres and neuritogenesis. Increasing the substrate elasticity, as well as decreasing the pattern width, increased the average length of neurites (Fig. 5a–b). Interestingly, the influence of substrate elasticity and geometric constraint are interchangeable. For instance, the reduction of neurite length in a soft substrate could be compensated by a narrow pattern, which enhanced neurite elongation. These results suggest that the effects of geometric constraint and substrate elasticity on neuronal differentiation are interrelated.

To further characterize the coupled effects of geometric constraint and substrate elasticity, the distributions of neurite length were also analyzed (Fig. 5c and d). For a wide pattern (e.g., 100 μm), the mean neurite length was small and exhibited a narrow distribution (Fig. 5c). As the pattern width decreased, the distribution widened and shifted from left to right, indicating enhanced neurite elongation. Similar effects, including longer average neurite length and wider neurite length distributions, were observed for neuronal spheres patterned on stiffer substrates (Fig. 5d). Interestingly, the neurite length distributions for stiff substrates and narrow patterns were not Gaussian and were better described by bimodal distributions. The peak values and fractions of cells in each peak were determined by bimodal curve fitting (Supplementary Fig. S2). The first and second peaks were approximately at 60 μm and 100 μm, respectively. As the cell pattern width became narrower or the substrates become stiffer, the cell populations at each peak shifted to the right slightly and the portion of cells in the second peak increased (Supplementary Table 1 and 2). The distribution of neurite length provides additional evidence on the interrelated mechanoregulatory effects of substrate stiffness and geometric constraint.

**Figure 6:**
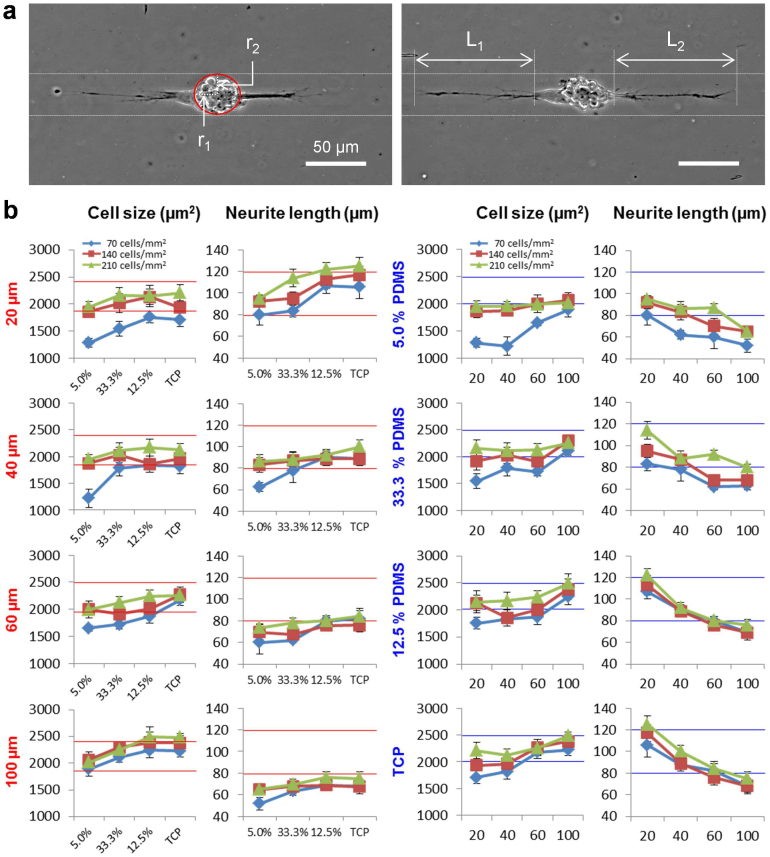
Effect of the cell seeding density on the cell size and neurite outgrowth of neuronal spheres. (a) Representative images of neuronal spheres. Average cell sizes and neurite lengths were extracted from the images. (b) Effects of the cell seeding density on the size of neuronal spheres and neurite lengths. The effects of the cell seeding density with different patterns (left) and substrate elasticities (right). Error bars represent standard deviations of 3 technical replicates.

The initial cell seeding density modulated the size of neuronal spheres, which influenced the cell-substrate mechanical interactions. The cell seeding density was therefore adjusted systematically to study the interrelated biomechanical effects of substrate elasticity and geometric constraint on neuronal differentiation (Fig. 6a). The cell seeding densities of 70, 140, and 210 cells/mm^2^, which formed stable neuronal spheres, were chosen. In this range, neuronal spheres with neurite outgrowth were formed along the patterns. As the cell seeding density increased from 70 cells/mm^2^ to 210 cells/mm^2^, the size of the neuronal spheres increased (Supplementary Fig. S3). The neuronal sphere dimension and the neurite length under different substrate elasticities, geometric constraints, and cell seeding densities were measured systematically (Fig. 6b). The dimension of neuronal spheres generally increased with the cell seeding density. Similar to narrow patterns or stiff substrates, high cell seeding density (i.e., large neuronal spheres) enhanced neurite elongation. The influence of the cell seeding densities on the neuronal sphere dimension and the neurite length was particularly noticeable on soft substrates. Similarly, the length of neurites was also more sensitive to the seeding density in narrow patterns. These results collectively support the notion that the influence of substrate stiffness and geometric constraint are interrelated and jointly regulate the differentiation of neuronal spheres.

### Computational biomechanical analysis

**Figure 7:**
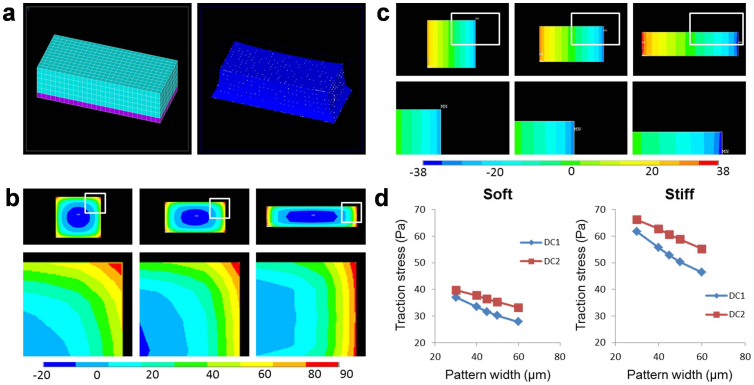
Finite element analysis of the effects of the geometric constraint, the cell size and substrate elasticity on cellular stress. (a) 3D finite element model for estimating cellular stress. (b) The first principal stress at the cell-substrate interface for different pattern widths (top). The stress distributions near the boundaries in regions indicted by the yellow rectangles in the top images (bottom). (c) Shear stress distribution in the long axis at the cell-substrate interface (top). The stress distributions near the boundaries in regions indicted by the yellow rectangles (bottom). Color bars represent cellular stress in Pascal. (d) The shear stress at the boundary for soft and stiff substrates. DC1 and DC2 represent 3600 and 4800 μm^2^ cell areas, respectively.

The coupled effects of geometric constraint, substrate elasticity, and cell size suggest that the differentiation of neuronal spheres is regulated mechanically. A finite element model was developed to elucidate the interrelationship between these factors on the cell-substrate mechanical interactions (Fig. 7a). In the simulation, neuronal spheres were confined in patterns with different widths while maintaining a constant total area. The distributions of the first principal stress at the cell-substrate interface are shown in Fig. 7b, top. For a constant area, cells confined in a narrower pattern were elongated and experienced a larger stress, especially at the boundary (Fig. 7b, bottom). The shear stress along the longest axis at the cell-substrate interface was also estimated to illustrate the geometric effect on the cell-substrate mechanical interactions along the pattern direction (Fig. 7c). Therefore, the geometric constraint enhanced the cellular stress induced by cell traction. The effects of other factors on the cell-substrate mechanical interactions, including the cell size and the substrate elasticity, were also investigated. Increasing the cell size, and therefore increasing the length of the longest axis, enhanced the cellular stress at the boundary. Similarly, a stiff substrate, which is known to increase the cell traction force^35^,^36^, enhanced the cellular stress at the cell-substrate interface (Fig. 7d). Taken together, the computational results support the notion that geometric constraint, substrate elasticity, and cell size control neuronal differentiation via the cell-substrate mechanical interactions.

## Discussion

In this study, plasma lithography patterned elastomeric substrates are reported to independently control the substrate elasticity, the geometric constraint, and the cell dimension for studying the influences of the mechanical interactions between the neuronal spheres and the substrates. Technologically, this study represents the first systematic investigation using plasma lithography to control substrate stiffness, physical confinement and cell size simultaneously. Plasma lithography is rapid (10 min), simple (one-step process), and effective (does not require extracellular matrix protein coatings). Compared to physical absorption and self-assembly monolayer-based patterning techniques, plasma lithography is based on direct surface functionalization and has excellent stability for long-term cell studies. In our experiments, neuronal spheres formed by neuroblastoma cells were confined in the patterns throughout the duration of the experiment (up to 15 days). Furthermore, patterning on flat elastomeric substrates facilitates the observation of neurite outgrowth and avoids topographic effects on cell differentiation. In the future, other elastomeric materials (e.g., other silicone gel) can be incorporated to replace PDMS for achieving an extended range of elasticity^37^.

Plasma lithography allowed us to study the effects of several mechanoregulatory factors on the differentiation of neuroblastoma cells and neuronal spheres. Once the cells were treated with retinoic acid, the differentiated cells could be clearly distinguished from undifferentiated cells due to glaring differences in their neurite outgrowth (see Supplementary Fig. S1). In agreement with reported studies^12^, the substrate elasticity enhanced the outgrowth of actin-rich neurites during retinoic acid-mediated differentiation of neuroblastoma cells (Fig. 2). In addition to the differentiation of individual neuroblastoma cells, our results further demonstrated that the differentiation of neuronal spheres depends on the substrate elasticity (Fig. 3). To investigate the effect of the substrate elasticity, 5.0%, 12.5%, and 33.3% PDMS with the elasticity of 0.7 MPa, 2.6 MPa, and 2.1 MPa respectively were chosen in this study. Remarkably, the differentiation of neuronal spheres correlated with the substrate stiffness, instead of the crosslinker concentration, supporting that the observed effects are not related to the crosslinker concentration or chemical properties of PDMS, but mediated mechanically. Furthermore, the size and shape of the neuronal spheres were controlled by geometric constraint and the cell seeding density independently. The results showed neuroblastoma cells in the neuronal sphere collectively regulated the outgrowth and distribution of neurites depending on the geometry of the neuronal spheres (Fig. 4,5,6). Our biomechanical analysis suggested the substrate elasticity, the geometric constraint, and the cell size collectively modulate the cell-substrate mechanical interactions to control neuronal differentiation (Fig. 7).

Analyzing the morphology of neuronal spheres revealed bimodal distributions of the neurite length, suggesting subpopulations of neuroblastoma cells may exist in the neuronal spheres. In fact, heterogeneity of neuroblastoma cells is well-documented in both tumors and tumor-derived cell lines^1^. Neuroblastic (N-type), substrate-adherent (S-type) and intermediate (I-type) cells have been characterized and these subtypes processed different differentiation and malignant potentials^38^. In particular, the I-type cells, which exhibit characteristics of caner stem cells, are subjected to intensive research. In our study, the bimodal distribution was particularly apparent for neuronal spheres on stiff substrates and narrow patterns. This observation may indicate the subtypes of neuroblastoma cells co-exist in the neuronal spheres and may have different mechanosensitivity. In conjunction with novel molecular probes and 3D imaging modularity^39^,^40^, the plasma lithography may represent a useful platform for elucidating their mechanosensitivity and relationship with cancer stem cells.

Our comprehensive techniques enable a systems approach for investigating the mechanoregulation of cells. Numerous studies have been reported on the effects on individual mechanical factors (e.g., matrix stiffness, shear stress, stretching, roughness and geometry) on cell behaviors. An outstanding challenge in the field is to understand the mechanoregulation from a systems perspective^19^,^41^. In this study, the interrelated effects of different factors can be understood collectively by the intercellular stress in the neuronal spheres as a result of the cell-substrate mechanical interaction. Computational biomechanical analysis was performed to elucidate the relationship between the geometry constraints, substrate stiffness, and cell density. In particular, the cellular stress due to the cell-substrate mechanical interaction can be increased by stiffer substrates, narrow pattern widths, and high cell seeding densities. The computational results are in good agreement with our experimental data. Importantly, our results highlight the importance of global mechanical structures when considering the mechanoregulation of biological systems and the influence of cell-substrate mechanical interactions. For instance, the mechanical traction force exerted on the surface by the migrating fronts of an epithelium is suggested to be a mechanical global entity^42^. Nevertheless, the exact molecular mechanisms by which multicellular structures sense and interpret the cell-substrate mechanical interactions remain poorly understood. Future investigation is warranted to clarify these issues. It is also possible that other mechanical factors, such as mechanical stretching, nanotopography, and fluid shear stress, can be incorporated into the platform to decipher the mechanoregulation of neuronal differentiation.

## Methods

### Preparation of PDMS substrates with different elasticities

PDMS (Sylgard 184, Dow Corning Corp., MI) was used as the substrate for cell culture. The Young's modulus of PDMS was controlled by the ratio between the crosslinker and the polymer base. A range of crosslinker concentrations, from 3.3% to 33.3%, was applied. After thorough mixing of the base and the crosslinker, the solution (3 g) was poured into a 60 mm polystyrene culture dish (VWR) and baked for 2 hours at 75°C. The elasticities of plasma-treated and untreated PDMS (1 mm thick) were measured by atomic force microscopy (5500 AFM, N9410S, Agilent, CA) with tapping mode tips (NSC 15, Nanosensors, Neuchatel, Switzerland)^43^.

### Plasma lithography

The PDMS template for plasma lithography was fabricated via photolithography of SU8 and PDMS molding. The template was placed on the substrate (0.5 mm thick) and exposed to atmospheric plasma (PDC-001, Harrick Plasma, NY) for 10 minutes. Due to physical shielding of the template, the substrate was selectively functionalized. A PDMS well was used to keep neuroblastoma cells in the patterned regions during seeding. The well was removed after allowing cells to attach for 3 hours. Retinoic acid (20.4 μL) was then added to the media (3 mL) to induce cell differentiation. The neuroblastoma cells were cultivated for 5 days and imaged.

### Cell culture

Human neuroblastoma cells, SH-SY5Y, were obtained from American Type Culture Collection (ATCC CRL 2266, VA). Cells were maintained at 37°C, 100% humidity and 5% CO2, and seeded at 25–50% confluence. The cells were cultured according to ATCC guidelines. Cell seeding densities from 30 cells/mm^2^ to 350 cells/mm^2^ were tested and used in the experiments.

### Immunostaining

The neuroblastoma cells were immunostained for F-actin with Alexa Fluor® 555 tagged phalloidin (Invitrogen, NY), vinculin for focal adhesions with monoclonal anti-vinculin antibodies conjugated with FITC (Sigma, MO), and nuclei with the ProLong® Gold antifade reagent containing DAPI (Invitrogen, NY). The cells were fixed with 3.7% formaldehyde (Polysciences, Inc., PA) for 10 minutes and permeabilized with 0.1% Triton X-100 (Astoria Pacific, OR) for 5 minutes. The cells were stained with phalloidin and anti-vinculin antibodies followed by the antifade reagents. A coverslip was placed over the cells and the edges were sealed with nail polish.

### Microscopy and image analysis

Bright-field images were captured using an inverted microscope (TE2000-U, Nikon) with a SPOT camera (Diagnostic Instruments model 2.2.1). Fluorescence images were captured using an inverted microscope (Leica model DMI4000B) with a Cooke SensiCam camera (The Cooke Corp., MI). The cell images were analyzed using NIH ImageJ to measure the size of neuronal spheres, the length of neurites, and the number of focal adhesions for each cell.

### Computational biomechanical analysis

A three-dimensional computational model was developed to analyze the cellular stress distribution of neuronal spheres using a commercial finite element package (ANSYS 13). In the simulation, the cell traction force was imposed by applying a uniform thermal strain computationally^44^. Three factors, namely pattern width, cell seeding density, and substrate elasticity were taken into account. To study the effects of geometric constraints, the cell area was considered constant for any seeding density. Cells with areas of 3600 or 4800 μm^2^ were confined in line patterns with 30, 40, 45, 50, and 60 μm widths. Two different thermal strains (as a result of 3 and 5°C temperature drops) were prescribed to the model to qualitatively represent the dependency of contractile force on the substrate elasticity^45^. To keep an equal mesh density in all cases, the element size was approximately 4 μm. The first principal stress and the shear stress along longest axis were calculated at the cell-substrate interface.

### Statistics

Statistical analysis was performed by Student's t-test and two-way ANOVA test followed by post-hoc Duncan test. *p* < 0.05 was considered statistically significant. Each data point represents an average of ~50 cells per each experiment from three independent experiments. Bimodal curve fitting was performed by OriginPro 8.5 with the multiple peak fit method to measure the distribution of the neurite length under different conditions.

## Author Contributions

K.H.N. and E.M. performed the experiments. N.J. implemented the finite element analysis. K.H.N., F.W., D.Z. and P.K.W. contributed to data analysis. K.H.N. and P.K.W. wrote the manuscript with comments and inputs from all authors.

## Supplementary Material

Supplementary Information Supplmentary Information
