# Supplementary material for: Probing Mechanoregulation of Neuronal Differentiation by Plasma Lithography Patterned Elastomeric Substrates
Source: Sci Rep. 2014 Nov 7;4:6965. doi: 10.1038/srep06965 (PMC4223667; doi:10.1038/srep06965)
Supplement: Supplementary Information — Supplmentary Information [file srep06965-s1.doc]

**Supplementary Information**

Probing Mechanoregulation of Neuronal Differentiation by Plasma Lithography Patterned Elastomeric Substrates

Ki-Hwan Nam^1,2^, Nima Jamilpour^1^, Etienne Mfoumou^1^, Fei-Yue Wang^3^, Donna D. Zhang^4^, and Pak Kin Wong^1,*^

^1^Department of Aerospace and Mechanical Engineering, The University of Arizona, Tucson, Arizona 85721, USA. Email: pak@email.arizona.edu; Tel: 520-626-2215; Fax: 520-621-8191

^2^Centre for Analytical Instrumentation Development, The Korea Basic Science Institute, Deajeon, 305-806, Korea

^3^The Key Laboratory for Complex Systems and Intelligence Science, The Institute of Automation, Chinese Academy of Sciences, Beijing, China.

^4^Department of Pharmacology and Toxicology, The University of Arizona, Tucson, Arizona, 85721, USA


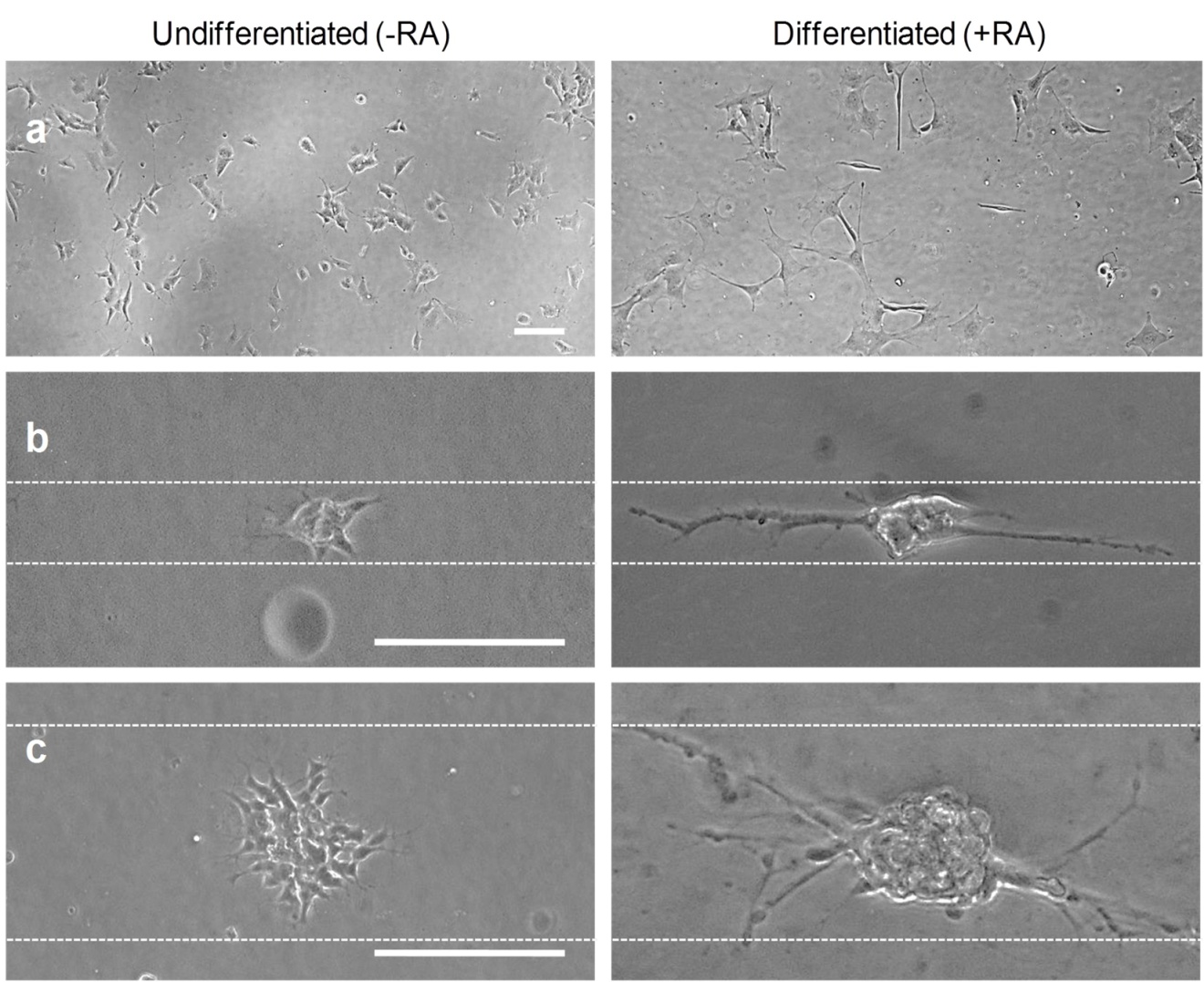


**Figure S1**│ **Undifferentiated (left) and differentiated (right) neuroblastoma cells induced by retinoic acid (RA)**. (a) Non-patterned neuroblastoma cells seeded on a plasma-treated 12.5 % PDMS substrate. (b) Micropatterned neuroblastoma cells on a polystyrene cell culture plate with 40 µm width, and (c) on a 12.5 % PDMS substrate with 100 µm width. The images were captured after 6-day culture. Scale bars, 100 µm.


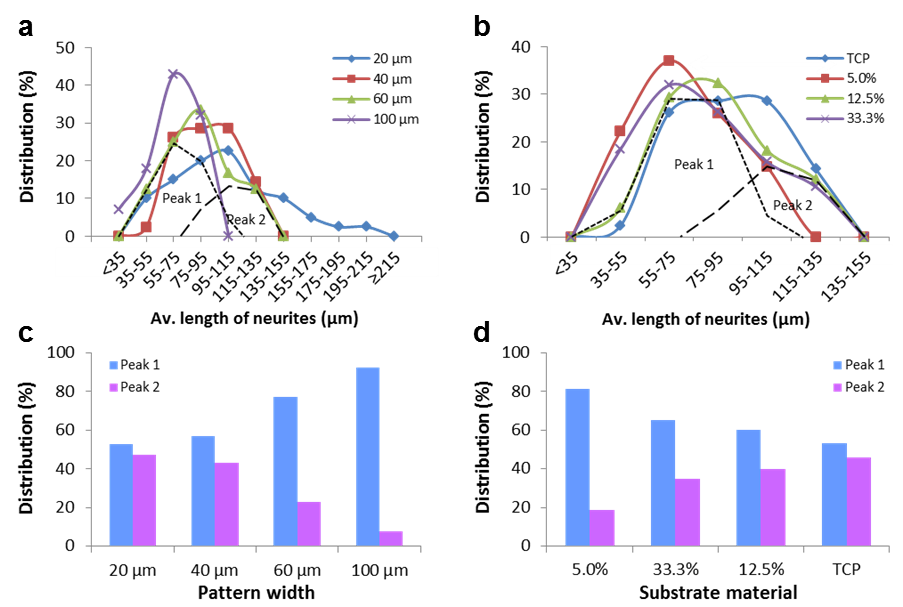


**Figure S2**│ The distribution of neurite length was analyzed for different pattern widths (a, c) and substrate materials (b, d). Dash lines indicate examples of the multiple peak analysis of 60 µm width (a) and 12.5% PDMS (b). The peaks and percentages are summarized in Table S1 and S2.


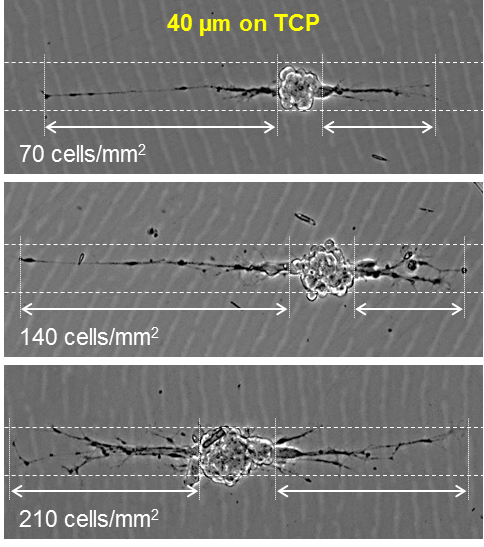


**Figure S3│** **Representative images of patterned neuronal spheres on tissue culture plates**. White dotted lines indicate 40 µm line patterns defined by plasma lithography and arrows indicate the extended neurite length.

**Table S1│** **Neurite length distribution with various pattern widths**.

|  | Peak 1 | | Peak 2 | |
| --- | --- | --- | --- | --- |
| Width [µm] | Length [µm] | % | Length [µm] | % |
| 100 | 60.0 | 92.5 | 85.0 | 7.5 |
| 60 | 72.1 | 77.4 | 104.3 | 22.6 |
| 40 | 5.5 | 57.1 | 109.4 | 42.9 |
| 20 | 83.0 | 52.9 | 140.1 | 47.1 |

**Table S2│** **Neurite length distribution with various substrate elasticities**.

|  | Peak 1 | | Peak 2 | |
| --- | --- | --- | --- | --- |
| Width [µm] | Length [µm] | % | Length [µm] | % |
| PDMS 5.0% | 63.0 | 81.5 | 96.4 | 18.5 |
| PDMS 33.3% | 64.6 | 65.1 | 105.9 | 34.9 |
| PDMS 12.5% | 72.6 | 60.0 | 108.3 | 40.0 |
| TCP | 76.5 | 53.3 | 111.6 | 45.7 |
